# Foreign Books for the Institutional Library

**Published:** 1913-02-15

**Authors:** 


### FOREIGN BOOKS FOR THE INSTITUTIONAL LIBRARY.

During the last five years various interesting books have been published in Germany and elsewhere dealing with matters of institutional interest. It is our endeavour in this article briefly to draw the attention of our readers to certain of the best volumes of this class of expert literature. We do so because some of these books deserve a wider and better recognition from English institutional workers than they have hitherto received. Perhaps the reason for the neglect that has fallen to them is to be sought in the fact that they are published in a foreign language, and that no one has so far deemed it worth while to translate them. But expert literature is international; it knows no one language or one style, and he who wishes to be an authority on his work must at least be acquainted with the best that has been written by other authorities in his special branch of interest.

Nor is this all. While many of these books are chiefly interesting because of their text and the written opinion that they disclose, there are some which are interesting mainly because they give us plans and diagrams, photographs and illustrations, which can be readily understood by the expert, even though he is not acquainted with the language or nomenclature used in their description. With us we have several such books. For example, some of our earlier works on hospital construction are now entirely out of date so far as their opinions are concerned, but the plans which they give have still an interest to those who are concerned in the building and fitting of new institutions. If we may refer to a lately published work, that of Dr. Mackintosh, we may say that in this, too, the principal value lies in the excellent illustrations with which the text is elucidated. We might draw further examples from our own and from foreign literature, but we think the above instance is sufficient to justify the conclusion that some books should not be neglected simply because they are written in a language which is unfamiliar to most of us. The proper institutional library should possess some of these newer works for purposes of reference, and the good librarian should make a point of adding to them from time to time by buying the best of the newer publications that see the light.

### France.

One of the best books on construction which have appeared during the past five years is Depage and Vandervelde's "Manual of Hospital Construction," which is printed in French and contains numerous excellent ground plans, detailed sketches, and, what is perhaps more interesting, "ideal plans" drawn on a scale which has been authorised by the Belgian Association of Architects. This is practically the only important book on hospitals published in French. Occasionally interesting articles dealing with hospital construction and describing new buildings appear in certain French technical papers. Of these the most important journals are the *Annales d'hygiène publique*, published at Paris, the *Journal d'hygiène*, the *Moniteur de l'hygiène publique*, and the *Mouvement hygiénique*, which is a monthly published in Brussels.

### Italy.

In Italy there have recently been some interesting developments in hospital construction, chiefly due to the reconstruction of the Rome, Naples, and Turin hospitals. While no special books dealing with these improvements have appeared, the journals devoted to hygiene and architecture have given descriptions of some of these new structures. Articles of interest will be found in the *Gazetta degli ospitali* of Milan, in the *Rivista d' igiene e sanità publica* of Turin, and in the Milan *Giornale della Reale Società Italiana d. igiene*. Nor must we omit to mention the excellent descriptive articles which have appeared in certain Russian papers, of which the fortnightly *Hygiene und Sanität* published at St. Petersburg is the chief, followed by the concise monthly *Moderne Medizin und Hygiene*. Both these periodicals are in the Russian language, with occasionally an article in German.

### Holland.

In Holland there was started two years ago a monthly periodical devoted solely to hospitals, and the files of this paper contain very instructive articles and plans of the newer Dutch hospitals. This paper is *Het Ziekenhuis*, which is the official organ of the Dutch Hospital Association. The Dutch *Tijdschrift voor Geneeskunde* and its contemporary, devoted to the interests of the medical profession in the Eastern Dutch colonies, have published some equally interesting articles which, owing to the fact that they are written in Dutch, are likely to escape the notice of the English reader. Unquestionably the most interesting special work in Dutch dealing with a hospital has been Dr. van Eysselsteen's description of the Groningen General Hospital. This is a sumptuous monograph, admirably illustrated with photographs and plans.

### Germany.

We now come to Germany, a country in which hospital progress has been remarkably rapid during the last ten years. Such progress has been accompanied by the publication of a large number of books and articles dealing with special or general subjects. Of these publications, the most important from all points of view is a book that has now become a classic, and that deserves to be translated into English. We refer, of course, to Dr. Grotjahn's well-known "Krankenhauswesen und Heilstättenbewegung," published by Vogel of Leipzig in 1908. This important work deals fully with the whole question of the economic, social, and medico-political aspects of hospitals and sanatoria. It is admirably and lucidly written, abounds in well condensed statistics, and gives valuable hints and suggestions that are worth consideration. It is one of the books that no institutional library should be without, for the problems with which it deals are ever present and are particularly pressing at this moment when we have the probable effects of National Insurance to consider. The first part deals with the impelling influences that account for the sudden and marked development of hospitalisation in Germany; herein are considered critically and expertly, the influence of advance in medicine, of social hygienic legislation, and of philanthropic progress. The second part deals with special and general hospitals, and gives full expositions of the progress of general, special, and private hospitals, of sanatoria, of cripple homes, of institutions for the blind, insane, idiots, epileptics, deaf and dumb persons, leprous patients, and chronic invalids. The third part discusses the two important questions "hospitalisation" for patients who can derive benefit from treatment, and asylum control for those who are incurable.

First and foremost, as representing to hospital workers in Germany and on the Continent generally what The Hospital and its back files represent to the English hospital worker, comes the *Zeitschrift für Krankenanstalten.* This is a weekly journal devoted to the discussion of hospital topics. It is the official organ of the *Vereinigung der leitenden Verwaltungsbeamten von Krankenanstalten Deutschlands* (Association of Controlling Officials of the German Hospitals), of the Society of the Hospitals of Westphalia and the Lower Rhine Provinces, and of the important and powerful Bavarian Hospitals Association, which is representative of the best hospital opinion abroad. The *Zeitschrift* is a highly technical and authoritative organ, which lacks to some extent the popular appeal that makes The Hospital attractive to the committeeman as well as to the expert; it is an expert journal written by experts for experts, and deals in technical fashion with the various questions which from time to time arise for discussion. A file of this useful journal contains articles on nearly every subject of interest to hospital workers; it gives full details of economic ventures, plans of new hospitals, and notes on construction, besides accounts of the various legislative measures which affect the German hospitals. Some of the most important articles on the German system have appeared in this journal, together with authoritative articles on technical subjects which can be thoroughly relied upon. Thus we may mention among others Dr. Rapp's exhaustive description of the hospital dispensary, with detailed statistical accounts of actual dispensaries in working; Herr Zeidler's equally interesting article on "Medical *versus* Lay Superintendents"; Oberinspector Einert's full account of the "Financial Relations of the German Hospitals" (recently referred to by Sir Henry Burdett); Professor Crede's summary of "The Essentials of Hospital Construction in Tropical Climates"; Architect Dr. Ruppel's papers on "Hospital Ventilation" and on "New Systems of Hospital Construction"; Inspector von Beatzin's paper on "Hospital Inventories"; Architect Inspector Böthke's paper on "The Building of Large Modern Hospitals"; and Architect Eitel's article on "The Modern Operating Theatre," besides many others of equally interesting and authoritative nature. The subscription to the journal is 16s. yearly, and Herr Leineweber, the publisher, informs us that complete files of the back numbers are still obtainable (Nos. I. to VII. only) at an average price of 16s. per bound volume.

The *Zeitschrift für Städtehygiene und Gesundheitstechnik*, published by the same firm, is a much older journal, which contains equally interesting and valuable articles, since it deals with hospital construction and building from 1875 to the present day and printed papers of considerable interest and merit. Complete sets of the back numbers are purchasable at prices varying from 16s. to 20s. per volume. These files are almost indispensable to those who wish to consult the original articles referred to in the text-books on hospital construction published in the decade 1888-1898.

Of the newer manuals, which deal more exclusively with hospital matters, the most important are those published by Leineweber, though some interesting works stand to the credit of the Fischer firm. Herr Zeidler's useful "Handbuch für Bau, Einrichtung, wirtschaftliche Betrieb, Organisation und Verwaltung von Kranken- und Pflegeanstalten" (issued at 7s. net) is a valuable compendium of essentials, and represents in Germany what Dr. Mackintosh's well-known book represents in this country. It gives some useful plans, and abounds in practical hints. The annual "Pocketbook" ("Taschenbuch . . . nebst Krankenanstalts Betriebs Lexikon," 4s. net) is under the direction of Herr Zeidler, and is published at the expiration of every financial year. It corresponds to our "Burdett's Hospitals and Charities," and gives valuable statistical information, arranged in an exceptionally able manner, concerning all the bigger hospitals in Germany; later editions have included details concerning the Austrian institutions as well. The information is authoritative and official and may be thoroughly relied upon.

Summary.

We have little space to deal with the remaining important books and a mere summary of names must suffice. Thus we may mention Dr. Lähr's "Die Anstalten für Psychische Kranke in Deutschland, Oesterreich, der Schweiz und d. Bältischen Ländern," published by Reimer, of Berlin, which gives an account of the newer institutions for the insane; "Bäder und Heilstätten-Lexikon," a special book-form supplement of the *Kranken-Journal*, published at Berlin; Herr Diesener's exhaustive "Betrieb, Einrichtung und Verwaltung der Krankenhäuser," published by Barth, of Leipzig, which is the supreme German authority—in some respects already a trifle out of date—on existing German institutions; Architect Ruppel's book on "Recent Hospital Construction"; Zeidler's "Adressbuch," which is a concise directory, with interesting short descriptions, of all the German hospitals; and the large number of new books, mainly legal, dealing with the relation between hospitals and the various Krankenkassen or Insurance Societies. The majority of these works, we may add, can be seen at the Library of the British Hospitals Association.

